# Supplementary material for: Splice-Junction-Based Mapping of Alternative Isoforms in the Human Proteome
Source: Cell Rep. Author manuscript; Available in PMC 2020 Jan 15. (PMC6961840; doi:10.1016/j.celrep.2019.11.026)
Supplement: 2 [file NIHMS1546469-supplement-2.zip › DF1/PXD010154/Liver/Liver_1_ACADSB_GVGSFCLSEAGAGSDSFALK.pdf]

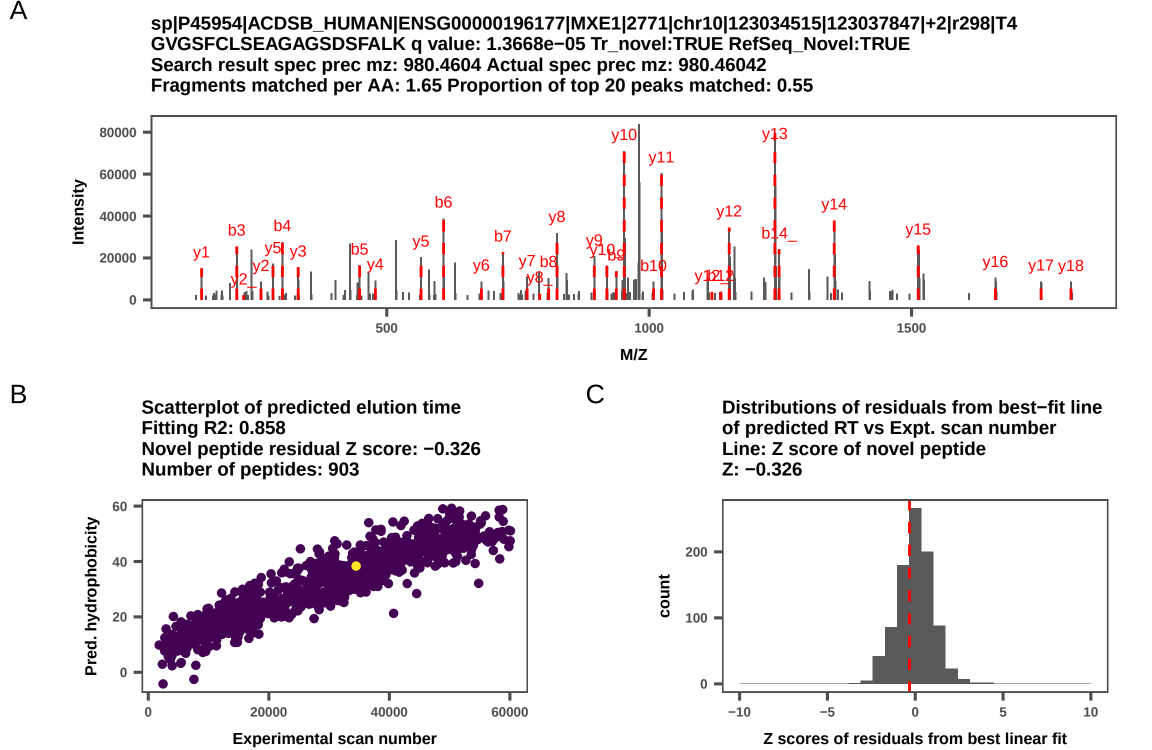
A
sp|P45954|ACDSB_HUMAN|ENSG00000196177|MXE1|2771|chr10|123034515|123037847|+2|r298|T4
GVGSFCLSEAGAGSDSFALK q value: 1.3668e−05 Tr_novel:TRUE RefSeq_Novel:TRUE
Search result spec prec mz: 980.4604 Actual spec prec mz: 980.46042
Fragments matched per AA: 1.65 Proportion of top 20 peaks matched: 0.55
Intensity
80000
60000
40000
20000
0
500
1000
1500
M/Z
y1
b3
b4
y5
y3
y2
y2_
b5
y4
y5
b6
y6
b7
y7
b8
y8_
y8
y9
y10
b9
y10
y11
b10
y12
b14_
y13
y14
y15
y16
y17
y18
B
Scatterplot of predicted elution time
Fitting R2: 0.858
Novel peptide residual Z score: −0.326
Number of peptides: 903
Pred. hydrophobicity
60
40
20
0
0
20000
40000
60000
Experimental scan number
C
Distributions of residuals from best−fit line
of predicted RT vs Expt. scan number
Line: Z score of novel peptide
Z: −0.326
count
200
100
0
−10
−5
0
5
10
Z scores of residuals from best linear fit
